# Inflammatory Gene Expression Upon TGF-β1-Induced p38 Activation in Primary Dupuytren's Disease Fibroblasts

**DOI:** 10.3389/fmolb.2015.00068

**Published:** 2015-12-08

**Authors:** Maro Bujak, Ivana Ratkaj, Elitza Markova-Car, Davor Jurišić, Anita Horvatić, Srđan Vučinić, Jonatan Lerga, Mirela Baus-Lončar, Krešimir Pavelić, Sandra Kraljević Pavelić

**Affiliations:** ^1^Division of Molecular Medicine, Ruer Bošković InstituteZagreb, Croatia; ^2^Department of Biotechnology, Centre for High-Throughput Technologies, University of RijekaRijeka, Croatia; ^3^Clinic for Surgery, Department for Plastic and Reconstructive Surgery, University Hospital Centre RijekaRijeka, Croatia; ^4^Faculty of Engineering and Centre for Advanced Computing and Modelling, University of RijekaRijeka, Croatia; ^5^Centre for Advanced Computing and Modelling, University of RijekaRijeka, Croatia

**Keywords:** inflammatory genes, myofibroblasts, p38 MAPK, MK2 kinase, extracellular-matrix

## Abstract

**Objectives:** Inflammation is an underlying mechanism behind fibrotic processes and differentiation of cells into myofibroblasts. Presented study therefore provides new data on activation of autoimmune and inflammatory immune response genes that accompany activation of p38 and cell differentiation in primary cells derived from Dupuytren's disease (DD) patients.

**Methods:** Primary non-Dupuytren's disease cells (ND) were isolated from macroscopically unaffected palmar fascia adjacent to diseased tissue obtained from patients diagnosed with the last stage of DD and cultured *in vitro*. Gene expression, collagen gel contraction assay and analysis of secreted proteins were performed in ND cells treated with TGF-β1 and/or inhibitor of p38 phosphorylation.

**Results:** During differentiation of ND fibroblasts, increased expression of immune response genes *PAI-1, TIMP-1, CCL11*, and *IL-6* was found. These changes were accompanied by increased cell contractility and activation of p38 and its target kinase MK2. Inhibition of p38 phosphorylation reversed these processes *in vitro*.

**Conclusions:** TGF-β1 induced p38 phosphorylation in ND cells grown from macroscopically unaffected palmar fascia adjacent to diseased tissue from DD patients. This was accompanied by activation of the cytokine genes *CCL-11* and *IL-6* and secretion of extracellular matrix regulatory proteins PAI-1 and TIMP-1. A combined approach directed toward inflammation and p38 MAPK-mediated processes in DD might be considered for improving management of DD patients and prevention of recurrence.

## Introduction

Fibrotic and fibroproliferative disorders may affect all tissues causing loss of tissue structure and function (Wynn, 2008). This process involves differentiation of cells into myofibroblasts accompanied by extracellular matrix deposition. Benign and malignant fibroproliferative disorders include idiopathic pulmonary fibrosis, hepatic cirrhosis, myelofibrosis, systemic sclerosis, Dupuytren's disease (DD), hypertrophic scars, and keloids (Huang and Ogawa, 2012). Common mechanisms occur in these fibrotic processes that include persistent inflammation and local overproduction/activation of different cytokines (Ghosh and Vaughan, 2012; Huang and Ogawa, 2012). For example, infiltrating immune cells in DD excrete large amount of different cytokines, chemokines and growth factors, especially TGF-β1 (Baird et al., 1993), cytokine that induces typical phenotypic changes and inflammation (Parsonage et al., 2005; Lupher and Gallatin, 2006). Under such conditions, cells may acquire myofibroblast phenotype. Myofibroblasts are active components of innate immune system and may regulate switches from acute to chronic inflammation (Kalluri and Zeisberg, 2006). In particular, activated myofibroblasts express MHC I and MHC II antigens (Brennan et al., 1990; Knittel et al., 1999) and respond to pro-inflammatory cytokines TNF-α and IFN-γ by releasing chemokine CCL2 (Marra et al., 1993), inflammatory mediators IL-1, IL-6, IL-8, prostaglandins and hyaluronate (Yellin et al., 1995; Sempowski et al., 1997; Schwabe et al., 2001). Moreover, during wound healing, phagocyte apoptotic cells and activate TGF-β1, a central mediator of fibrosis (Canbay et al., 2003). Molecular mechanisms underlying fibroproliferative disorders have been studied for years by different approaches including modern large-scale methodologies but effective treatments for this group of disease are still missing. For example, surgery is still the only available option for DD patients even though high recurrence rates are usually observed. Novel potential therapeutic targets for DD and fibroses have therefore, been studied, including reactive oxygen species-dependant TGF-β signaling (Samarakoon et al., 2013), focal adhesion kinases (Lagares and Kapoor, 2013), and inflammatory cytokine TNF (Verjee et al., 2013). In the present paper, we analyzed the expression of pro-inflammatory cytokine genes in primary cells grown from DD patients and found that TGF-β1 induced p38 phosphorylation in ND cells grown from macroscopically unaffected palmar fascia adjacent to diseased tissue from DD patients is accompanied by activation of the cytokine genes CCL-11 and IL-6 and secretion of extracellular matrix regulatory proteins PAI-1 and TIMP-1, which casts new light on pathogenesis of DD.

## Materials and methods

### Primary cell cultures

Clinical specimens were collected in strict compliance with the clinic's chief pathologist and the ethics committee for research involving human subject at Clinical Hospital “University Hospital Centre Rijeka” in Croatia. Informed consent was signed by all patients. The cells were isolated according to established protocols (Tse et al., 2004; Pavelic et al., 2009). Primary ND cells were obtained from macroscopically unaffected palmar fascia adjacent to the disease tissue. Tissue samples were collected from six patients (age range 54–75 years, males) diagnosed with the last stage of DD. Second passages were used for all experiments to assure uniformity and prevent loss of original cell phenotype. ND cells were cultured as monolayers and maintained in Dulbecco's modified Eagle medium (DMEM, Gibco, Invitrogen, USA) supplemented with 10% fetal bovine serum (FBS, Gibco, Invitrogen, USA), 2 mM L-glutamine (Gibco, Invitrogen, USA), 100 U/mL penicillin (Gibco, Invitrogen, USA), and 100 μg/mL streptomycin (Gibco, Invitrogen, USA) in a humidified atmosphere with 5% CO2 at 37°C until they reached 80% confluence.

### Western blotting

ND cells were treated with TGF-β1 or SB203580 inhibitor and TGF-β1 for 16 h. Cells were lysed in the buffer containing 50 mM Tris HCl (pH 8), 150 mM NaCl, 1% NP-40, 0.5% sodium deoxycolate, 0.1% SDS, protease inhibitor cocktail (Roche, Basel, Switzerland) and phosphatase inhibitor cocktail (Thermo Scientific, Waltham, Massachusetts, USA). Protein concentration was determined with DC Protein Assay Kit (BIO-RAD, USA) and a total of 40 μg of proteins were resolved on 9% polyacrylamide gels using the Mini-protean cell (Bio-Rad, Foster City, CA, USA), and analysis was performed by previously established procedure (Pavelic et al., 2009). The membranes were incubated with primary antibodies raised against phosphorylated MAPKAPK-2 (MK2; 1:1000, phospho-MAPKAPK-2, Cell Signaling technology, Danvers, Massachusetts, USA) at 4°C overnight. Secondary antibody linked to anti-rabbit (1:1300, Dako, Denmark) horseradish peroxidase was used. The signal was visualized by Western Lightening Chemiluminescence Reagent Plus Kit (Perkin Elmer, Waltham, Massachusetts, USA) on the VersaDoc Imaging System 4000 (Bio-Rad, Foster City, CA, USA). α-tubulin was used as a loading control (1:1000, Sigma-Aldrich, St. Louis, Missouri, USA).

### Expression analysis of cytokines and cytokine receptors by PCR array

For detection of gene expression involved in cytokine inflammatory and autoimmune response pathway-focused RT^2^ Profiler PCR Array: Human Inflammatory Response and Autoimmunity (PAHS-077A, SABiosciences, Valencia, CA, USA) was used. Analysis was conducted according to manufacturer recommendations. confluent ND cells (80%) were deprived of serum for 2 h prior to treatment with TGF-β1 (3 ng/mL in serum free medium, R&D Systems, McKinley Place, MN, USA) and TGF-β1 together with the inhibitor of p38 phosphorylation SB203580 (10 μM, Calbiochem, Darmstadt, Germany). In dual treatment, cells were first incubated with inhibitor SB203580 for 30 min and then stimulated with TGF-β1. After 16 h total RNA was isolated using RNAeasy spin columns (Qiagen, Valencia, CA, USA). First strand cDNA synthesis reaction mix containing RT cocktail was incubated with genomic DNA elimination mixture at 42°C for 15 min and afterwards immediately heated at 95°C for 5 min. To each of cDNA synthesis reactions, the RNase-free water was added up to 102 μl and the samples were stored overnight at −20°C. A total of 25 μl of prepared experimental cocktail was added to each well of PCR array. Thermal cycling conditions consisted of initial denaturation step, 1 cycle of 95°C for 10 min, 40 cycles of 95°C for 15 s and annealing and extension at 60°C for 1 min. For data analysis, free PCR array data analysis web portal was used (http://www.SABiosciences.com/pcrarraydataanalysis.php).

### Protein fractionation by sodium dodecyl sulfate polyacrylamide gel electrophoresis (SDS-PAGE)

After 16 h, conditioned media (20 ml) where ND cells treated in the same manner as cell used for expression analysis were grown, was collected on ice, centrifuged at 4°C by 1500 rpm for 15 min to remove cells and debris and supplemented with complete protease inhibitor cocktail (Roche, Basel, Schwitzerland). The samples were concentrated by ultra-filtration (Millipore, Billerica, MA, USA, Centricon, 3 kDa cut-off) at 4°C by 12,000 rpm. Concentrated samples were dissolved in a buffer containing 7 M urea, 2 M thiourea, 4% CHAPS, 0.2% Bio-Lyte ampholyte pH 3–10 (Bio-Rad), 1% DTT and 1X protease inhibitor cocktail (Roche, Basel, Schwitzerland). The protein concentration was determined by Bradford Assay (Bio-Rad, Foster City, CA, USA) as described previously (Pavelic et al., 2009).

### One-dimensional polyacrylamide gel electrophoresis

One-dimensional polyacrylamide gel electrophoresis (SDS-PAGE) was performed on a Bio-Rad mini-Protean system. A total of 40 μg of proteins were resolved on the 12% one-dimensional polyacrylamide gel. Gels were stained with Coomassie Brilliant Blue G-250 (Bio-Rad, Foster City, CA, USA) and scanned by VersaDoc Imaging System 4000 (Bio-Rad, Foster City, CA, USA). Qualitative image analysis was carried out using PDQuest SW, 7.0 (Bio-Rad, Foster City, CA, USA).

### Trypsin digestion and mass spectrometry

The lanes were manually excised into 24 bands (eight bands per lane) which were processed for tryptic digestion according to standard protocol (Shevchenko et al., 2006). Briefly, after destaining step with 40% methanol/10% acetic acid, gel plugs were incubated with 50 mM ammonium bicarbonate for 10 min at 37°C and then subsequently incubated with 50% ammonium bicarbonate/50% acetonitrile (ACN) at 37°C. After incubation with 50% acetonitrile for 5 min at 37°C the proteins were digested with 10 ng/μl of trypsin in ammonium bicarbonate overnight at 37°C. Upon completion and digestion, 0.1% TFA was added per well. Each tryptic peptide mixture thus generated was concentrated, desalted with C_18_ ZipTip (Millipore, Billerica, MA, USA), and subsequently eluted with 4 mg of cyano-4-hydroxycinnamic acid in 60% acetonitrile and 0.1% trifluoroacetic acid onto MALDI sample plate. The samples were analyzed on the 4800 Plus MALDI TOF/TOF analyzer (Applied Biosystems Inc., Foster City, CA, USA) equipped with Nd:YAG laser operating at 200 Hz. All mass spectra were recorded from 750 to 4000 Da in positive reflectron mode. MS spectra were recorded for all sample spots on the plate and internally calibrated using signals from auto-proteolytic fragments of trypsin. Up to three spectral peaks per spot that met threshold criterion were included in the acquisition list for the MS/MS spectra analysis. Peptide fragmentation was performed at collision energy of 1 kV and collision gas pressure of ~2 × 10^−7^ Torr. MS and MS/MS spectra generated were searched against the Swiss-Prot and NCBI databases, respectively, using the Mascot software (Matrix Science, Boston, MA, USA). Monoisotopic peptide masses were used for combined MS and MS/MS database search in order to obtain protein identities. Following search criteria were applied: maximum allowed peptide mass error 20 ppm; fragment mass tolerance, ±0.3 Da; minimum 5 S/N; and a maximum of one incomplete cleavage per peptide. Only significant hits defined by the Mascot scoring and probability analysis (*P* = 0.05) were accepted as significant results.

### qRT-PCR

We tested expression of *PAI-1* and *TIMP-1* genes using SYBR Green-based qPCR. Total RNA was isolated from ND cells according previously described experiment for RT^2^ Profiler PCR Array and was reversely transcribed into cDNA by High Capacity cDNA Reverse Transcription Kit (Applied Biosystems) on GenAmp PCR System 2400 (Applied Biosystems). Primer pairs were designed by the software “Real Time PCR Tool” from Integrated DNA Technologies (http://eu.idtdna.com/scitools/Applications/RealTimePCR/). Designed primers were tested for specificity to give only one band (confirmed by gel electrophoresis and melting curve analysis) and conditions of reactions were optimized so that efficiency of PCR reaction was 95–100% (Table 1). Relative quantification values were obtained from the threshold cycle number of analyzed genes measured in triplicate and normalized with one most stable control gene using geNorm program. As control gene s18 *rRNA* was employed. Results were processed with REST 2008 V2.0.7.

**Table 1:** **Primers used for the qRT-PCR analysis**.

**Gene**		**Sequence 5′–3′**	**Size**	**Conditions**
18SrRNA	For	GTAACCCGTTGAACCCCATT	151 bp	60°C, 3.5 mM
	Rev	CCATCCAATCGGTAGTAGCG		MgCl_2_
PAI-1	For	TGGAACAAGGATGAGATCAG	262 bp	60°C, 3 mM
	Rev	CCGTTGAAGTAGAGGGCATT		MgCl_2_
TIMP-1	For	AGGCTCTGAAAAGGGCTTCCA	150 bp	60°C, 3 mM
	Rev	GAGTGGGAACAGGGTGGACA		MgCl_2_

After initial denaturation at 95°C for 3 min, 40 cycles of PCR were performed in AB 7300 Real-Time PCR System (Applied Biosystems, Foster City, CA, USA). Each cycle included denaturation at 95°C for 1 min, annealing at 60°C (see Table 1) for 30 s, and extension at 72°C for 10 s, followed by melting curve program.

### Collagen gel contraction assay

Collagen gels were prepared as described by Ngo et al. (2006). Briefly, fibroblasts were re-suspended in 400 μl DMEM supplemented with 0.2% FCS and added to 200 μl collagen suspension (3 mg/ml) yielding the final concentration of 150 000 cells/ml and 1 mg/ml collagen. Cell suspension containing 500 μl collagen was cast into each well of 24-well tissue culture plate and incubated at 37°C for 1 h in order to facilitate polymerization. After gelation, the gels were released from the surface of the culture well using a sterile tip. Gels were pre-incubated with synthetic p38 phosphorylation inhibitor SB203580 for 1 h. After stimulation with TGF-β1 (3 ng/mL) for 24, 48, and 72 h in the presence or absence of SB203580, contraction was digitally photo-documented at 24, 48, and 72 h. Contraction quantification was performed using NIH image software (http://rsb.info.nih.gov/nih-image/).

## Results

### Inflammatory and autoimmune gene expression in differentiating primary DD cells is linked with the activation of p38 MAPK signaling

**Figure 1:**
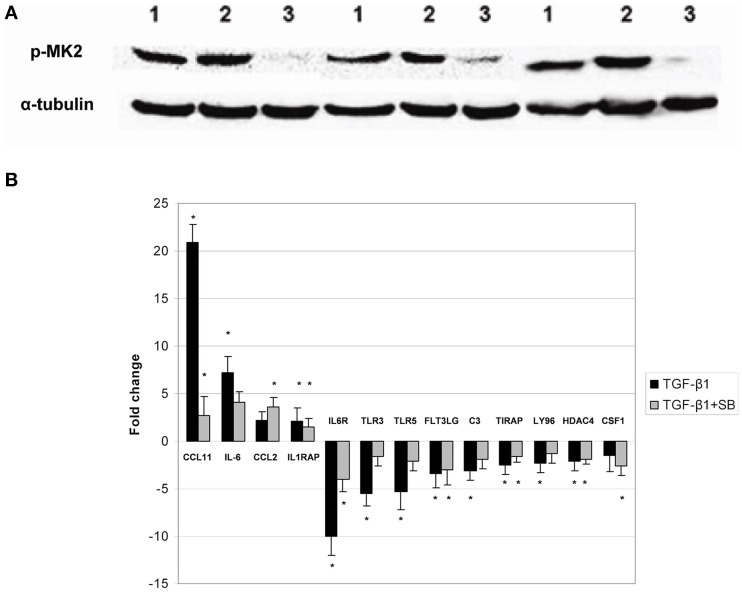
**Phosphorylation status of MAPKAPK-2 kinase and expression of cytokine and cytokine receptors in ND cells treated with TGF-β1 or TGF-β1 and inhibitor of p38 phosphorylation**. Panel **(A)** representative western blots showing phosphorylated MAPKAPK-2 in untreated ND cells (1), ND cells treated with TGF-β1 (2) and ND cells incubated with TGF-β1 and p38 phosphorylation inhibitor SB203580 (3), expression of α-tubulin in ND cells was used as loading control; Panel **(B)** changes in gene expression observed in ND fibroblasts treated with TGF-β1 (black columns) or treated with TGF-β1 and p38 phosphorylation inhibitor SB203580 (gray columns). The results are presented as gene expression fold change between treated cells and control. ^*^Statistically relevant changes (*p* < 0.05) in comparison to control.

We previously reported on the involvement of p38 MAPK signaling pathway in primary cells grown from DD patients (Ratkaj et al., 2012). In the present paper, we studied p38 MAPK downstream target, specifically MK2 kinase, during differentiation of primary ND cells into myofibroblasts. This kinase is exclusively phosphorylated by activated p38 and was previously implicated in myofibroblast differentiation and fibrotic processes other than DD (Liu et al., 2007). We detected stable endogenous expression of phosphorylated MK2 form in cells grown from macroscopically unaffected palmar fascia adjacent to diseased tissue (ND cells) prior and upon treatment with TGF-β1 (Figure 1A). However, in cells co-incubated with TGF-β1 and p38 phosphorylation inhibitor, the levels of phosphorylated MK2 diminished. Interestingly, active form of MK2 was detected in untreated ND fibroblasts as well, which could be attributed to the well-known role of p38 MAPK signaling in homeostasis of palmar fascia fibroblasts or in the intrinsic predisposition of normal palmar fibroblasts in DD patients for disease development. Since MK2 is involved in the regulation of inflammatory response within p38-MAPK signaling pathway (Gaestel, 2013), we analyzed changes in the expression profile of genes involved in inflammation and autoimmune response during the differentiation process. Obtained results showed that activation of p38 MAPK signaling by TGF-β1 in ND cells directly influences expression of a number of tested genes (Figure 1B) including genes encoding for chemokine (C-C motif) ligand 11 (*CCL11*), interleukin 6 (*IL-6*), chemokine (C-C motif) ligand 2 (*CCL2*), and interleukin 1 receptor accessory protein (*IL1RAP*) that were found to be over-expressed. Inhibition of p38 phosphorylation partially reversed this process and significantly down-regulated genes *CCL11* and *IL1RAP*. Similar down-regulation was also observed for *IL-6*. A number of genes had lower expression levels in ND cells activated by TGF- β1 including those coding for complement component (*C3*), fms-related tyrosine kinase 3 ligand (*FLT3LG*), interleukin 6 receptor (*IL6R*), lymphocyte antigen 96 (*LY96*), toll-interleukin 1 receptor (*TIR*) domain containing adaptor protein (*TIRAP*), toll-like receptor (*TLR3*), toll like receptor 5 (*TLR5*), and histone deacetylase 4 (*HDAC4*). Majority of the genes whose expression levels were altered by TGF-β1 showed reversed expression pattern upon treatment with the inhibitor of p38 phosphorylation. Obtained results thus support previous studies reporting on p38 MAPK pathway involvement in inflammation processes during fibroblast differentiation. Since myofibroblast-like phenotype includes initiation of extensive extracellular matrix deposition driven by inflammatory factors such as for example TGF-β1 and TNF-α (Reeves and Friedman, 2002), we profiled secretome of ND cells.

### Analysis of proteins secreted by ND cells

**Figure 2:**
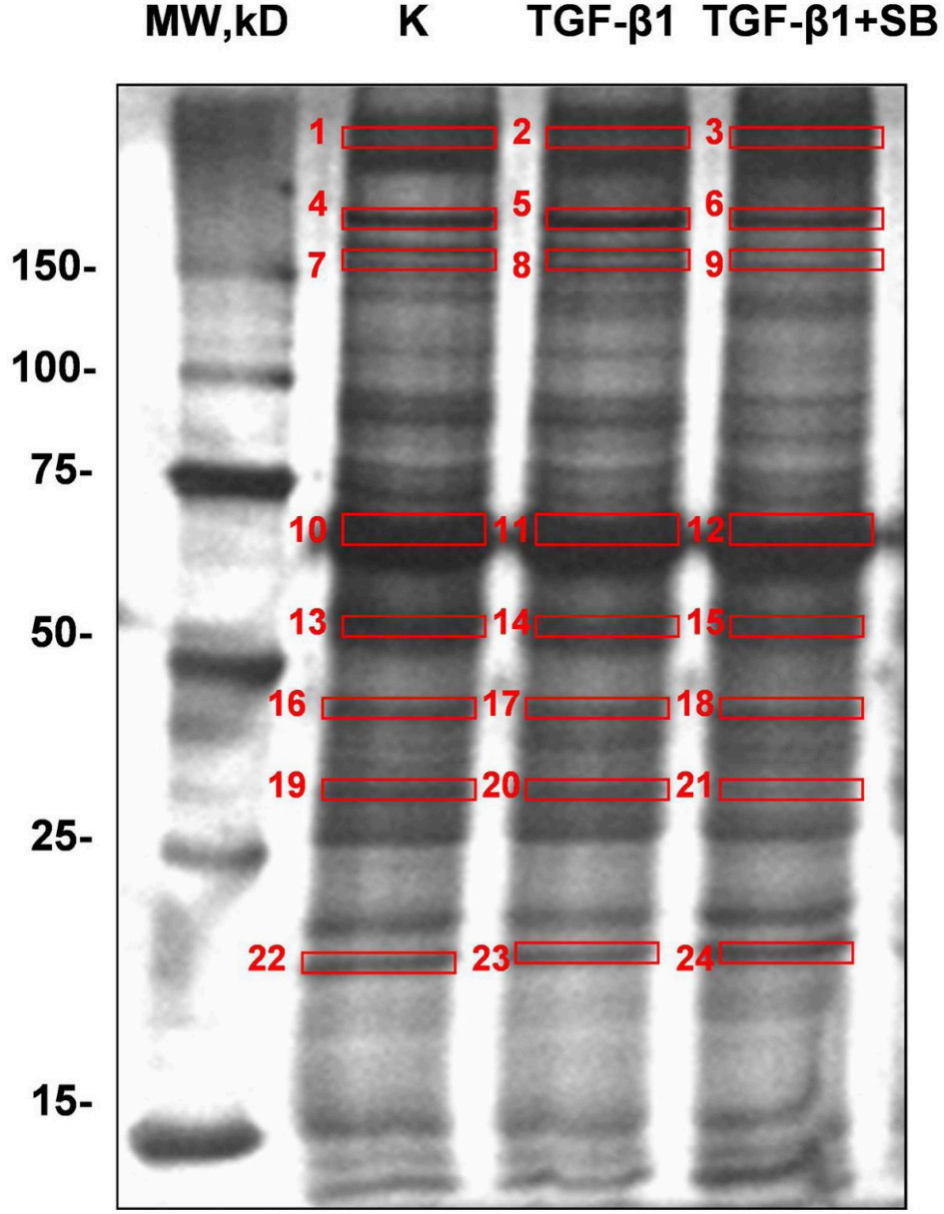
**Representative SDS-PAGE gels (12% bis-Tris gel) with proteins secreted into the cell growth medium by ND cells treated with TGF-β1 or with a combination of TGF-β1 and p38 phosphorylation inhibitor SB203580 under serum-free conditions**. Numbers denote bands with proteins which were excised, digested, and analyzed by mass spectrometry.

**Figure 3:**
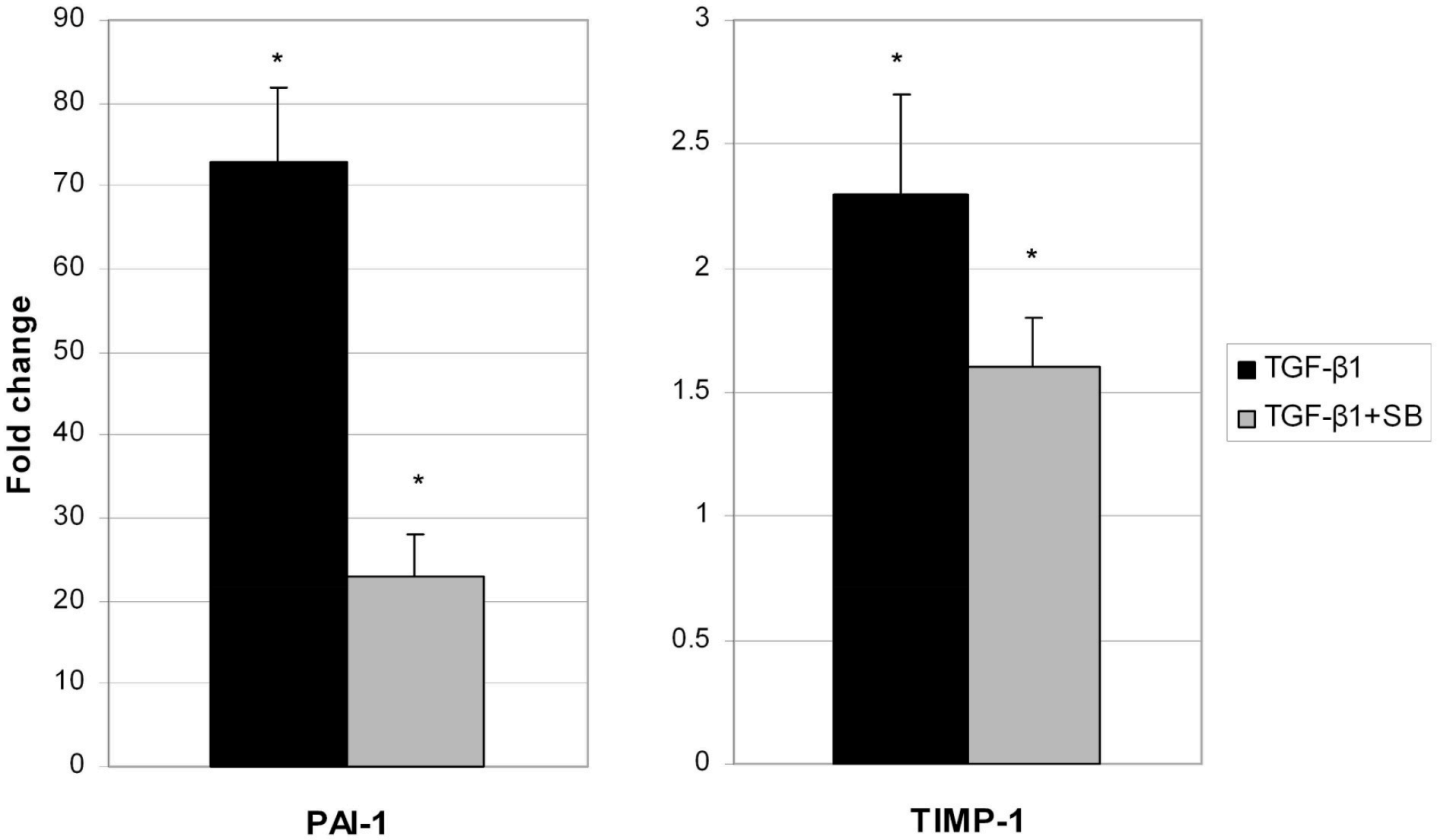
**Changes of PAI-1 and TIMP-1 gene expression in ND cells treated with TGF-β1 (black columns) or with TGF-β1 and p38 phosphorylation inhibitor SB203580 (gray columns)**. ^*^Statistically relevant changes (*p* < 0.05) in comparison to control.

We analyzed all secreted proteins (the secretome) from untreated (control), TGF-β1-activated ND cells and ND cells treated with TGF-β1 and inhibitor of p38 phosphorylation (Figure 2). Differentially expressed proteins are presented in Table 2. Extracellular matrix proteins were expectedly identified as a major group of secreted proteins. Incubation with TGF-β1 alone or in combination with the p38 phosphorylation inhibitor induced expression of matrix metalloproteinase-2 (MMP-2), collagen alpha-5(VI) chain (COL4A5), plasminogen activator inhibitor 1 (PAI-1), and tissue inhibitor of matrix metalloproteinases 1 (TIMP-1), in comparison with control cells (Table 2). TIMPs and indirectly PAI-1 are key inhibitors of MMPs and other enzymes that degrade ECM proteins. Their expression was validated at the gene level as well (Figure 3). Addition of p38 phosphorylation inhibitor significantly decreased changes induced by TGF-β1 treatment (diminished expression of *TIMPs* and *PAI-1*genes). Ulrich et al. (2009) showed that *TIMP-1* is the only TIMP whose expression is increased significantly in the DD nodule in comparison with normal palmar fascia. Surprisingly, until now there was no evidence on *PAI-1* gene expression in fibroblasts activated by TGF-β1 from palmar fascia.

**Table 2:** **Proteins expressed in the cell growth medium of ND cells**.

**Position on the gel**	**Protein name**	**Accession number/ncbi**	**MW**	**Treatment**	**Protein score (CI %)**
1,2,3	Fibronectin 1/FN1	gi|119590953	137,642	Control, TGFβ, TGFβ+ SB	100
5	Pro alpha 1(I) collagen COL1A2	gi|186893270	138826.6	TGFβ	100
8,9	Collagen, type II, alpha 1/COL2A1	gi|119578373	131077.4	TGFβ, TGFβ+ SB	100
11,12	Matrix metalloproteinase-2/MMP-2	gi|5822007	70872.2	TGFβ, TGFβ+ SB	100
14,15	Collagen alpha-5(VI) chain COL4A5	gi|183583553	279755.7	TGFβ, TGFβ+ SB	100
16,17	Matrix metalloproteinase 1/MMP1	gi|54697154	53947.7	Control, TGFβ	100
20,21	Plasminogen activator inhibitor −1/PAI-1	gi|755747	143389.2	TGFβ, TGFβ+ SB	100
23	Tissue inhibitor of matrix metalloproteinases 1/TIMP-1	gi|57210052	12526.5	TGFβ	100

*(1) Control ND cells (untreated cells), (2) ND cells treated with TGF-β1 (3 ng/ml), and (3) ND cells treated with TGF-β1 (3 ng/mL) and p38 phosphorylation inhibitor SB203580 (10 μM). Proteins were resolved on SDS-PAGE, cut from the gel, digested, and identified by mass spectrometry*.

### Collagen gel contraction assay

**Figure 4:**
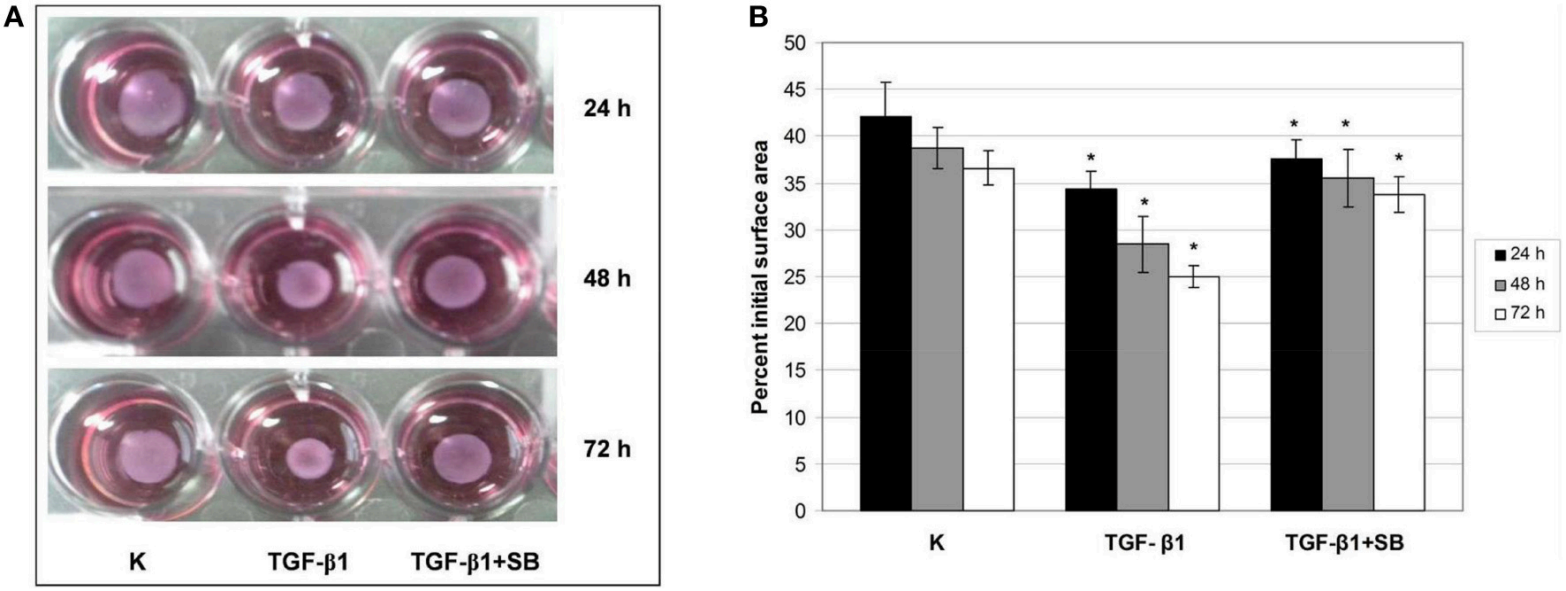
**TGF-β1–mediated contraction of ND fibroblast-populated collagen lattices is p38-dependent**. ND cells were seeded in neutralized collagen solution and treated with TGF-β1 or with a combination of TGF-β1 and p38 phosphorylation inhibitor SB203580 for 24, 48, and 72 h. Gels were detached, the gel contraction was digitally photo-documented **(A)** and measured as a reduction of gel surface 24 (black columns), 48 (gray columns), and 72 h (white columns) after detachment **(B)**. ^*^Statistically significant changes (ANOVA, *p* > 0.05).

Our previous results (Pavelic et al., 2009; Ratkaj et al., 2012) showed a connection between p38 MAPK activation and differentiation of fibroblasts into myofibroblasts and acquirement of their contractive phenotype. In the absence of an animal model to study the effect of p38 phosphorylation inhibition in DD cells during differentiation process, we used three-dimensional collagen lattice where the cells were grown on the mouse collagen type I layer. Type I collagen is abundantly present in healthy palmar fascia and therefore suitable to study fibroblast contraction during differentiation into myofibroblasts. Obtained results are presented in Figures 4A,B. TGF-β1 treatment significantly increased the contractility of cells after 24-h treatment reducing the gel size to 34% of its initial size compared with reduction of 42% in the control group. After 48-h incubation with TGF-β1, gel size was reduced to 29% of the initial surface in comparison with control reduced to 39%. Incubation of cells with TGF-β1 for 72 h decreased the surface of the gel to 25% of the initial surface in contrast to the control with observed 37% initial area decrease. Addition of p38 phosphorylation inhibitor before TGF- β1 stimulation blocked observed cell contraction. Thus, after 24-h treatment, gel surface was reduced to 38% of the initial surface area, after 48 h to 36% and after 72 h reduction was 34% of the initial surface. These results support the role of p38 activation in contractility of fibroblasts and their differentiation into myofibroblasts in the palmar region of DD patients.

## Discussion

Phenotypic changes during differentiation of fibroblasts into myofibroblasts are promoted by cytokines/chemokines and other immunomodulators (Lupher and Gallatin, 2006). This process is a persistent, yet localized inflammation that causes deposition of ECM components. Same processes are driven by TGF-β1 in DD (Krause et al., 2011). TGF-β1 protein family members transmit signals through type I and type II serine/threonine kinase receptors that further transduce the signal into the cytoplasm through phosphorylation of receptor-regulated Smad proteins. Activated R-Smads then regulate gene expression, i.e., expression of plasminogen activator inhibitor 1 gene (*PAI-1*), observed in the presented study as well (Table 2; (Krause et al., 2011)) and/or mitogen-activated protein (MAP) kinase signaling pathway genes (Figure 1; Moustakas and Heldin, 2005; Ratkaj et al., 2012). A number of papers describe the role of TGF-β1 in increased contractive properties of Dupuytren-derived cells (Wong and Mudera, 2006; Bisson et al., 2009). It has a role in induction of genes coding for ECM components as well, such as for example elastin, myelin basic protein and thrombospondin-1 (Ratkaj et al., 2012). In the presented study we analyzed cytokines and chemokines induced by TGF-β1 in primary derived fibroblast from unaffected palmar tissue of DD patients. Upon treatment, the most prominently over-expressed inflammatory gene was chemokine 11 (*CCL11*). Expression levels of *CCL11* diminished upon inhibition of p38 kinase phosphorylation (Figure 1B). Even though the role of chemokine CCL11 (eotaxin-1) was already described in proliferation of lung and bronchial fibroblasts (Rokudai et al., 2006), angiogenesis, expression of MMP-2, and the synthesis of type I collagen, its role was not described in cell contraction and differentiation processes (Huaux et al., 2005; Puxeddu et al., 2006). Therefore, CCL11 might be involved in the early phases of fibroblast differentiation when it induces up-regulation of several fibrogenic factors or chemokines (Tomasek et al., 2002), where other factors, i.e., TGF-β1 are required for further progression of this process. Furthermore, a role for CCL11 may be in triggering migration of progenitor mast cells toward the inflamed area and their activation during fibrotic changes (Price et al., 2003). Indeed, increased amount of mast cells was already observed in DD where they might actively participate in DD pathogenesis with CCL11 (Schubert et al., 2006). Additionally, it has been found that fibroblasts express CCR3 receptor that specifically binds the CCL11 ligand. Activated CCR3 probably induces fibroblasts proliferation and differentiation during fibrosis in an autocrine manner (Huaux et al., 2005). However, we were unable to detect *CCR3* gene transcripts, meaning that it is either not expressed in palmar fibroblasts or its expression occurs later on during disease progression (Hogaboam et al., 1998; Huber et al., 2000). Altered gene expression level in ND cells treated with TGF-β1 was observed for *IL-6* gene as well. IL-6 is cytokine produced by fibroblasts where it contributes to the development of fibrosis and inhibition of fibroblasts' apoptosis (Bunker et al., 2000; Liu et al., 2007; Genotyping results for *IL-6* gene are presented in Supplementary Material). IL-6 is a profibrogenic cytokine and its expression was already well-documented in different fibrotic diseases (Seong et al., 2009). Interestingly, primary fibroblasts grown from unaffected palmar fascia of DD patients express the *IL-6R* gene as well. Therefore, IL-6 trans-signaling promotes release of IL-6 from fibroblasts and endothelial cells in a positive autocrine feedback system, which is probably the case in our model as well.

Alteration in inflammatory genes observed in this study was accompanied by a number of proteins secreted in the cell growth media with a known role in ECM remodeling. These include matrix metalloproteinase 2 (MMP-2), tissue inhibitor of matrix metalloproteinase 1 (TIMP-1), and plasminogen activator inhibitor 1 (PAI-1) proteins. Overexpression of *PAI-1* and *TIMP-1* was validated on the gene level as well (Figure 3). Their transcripts levels diminished upon p38 phosphorylation inhibition. Similar results were reported by Leivonen et al. (2013) who identified p38 and ERK 1/2 as the key signal transducers of TGF-ß/Smad 3-induced overexpression of TIMP-3. They also found an overexpression of TIMP1 and PAI-1 after treatment with TGF-ß, whose influence was attenuated with p38 inhibitor. Increased amounts of PAI-1 have also been described in other fibroproliferative diseases as well (Sisson and Simon, 2007; Wang et al., 2007). Moreover, Tuan et al. (2008) showed that treatment of keloid fibroblasts with PAI-1 specific polyclonal antibody or siRNA leads to decreased synthesis and deposition of the ECM. To our knowledge, the relationship between TGF-β1 and PAI-1 has not yet been described in DD tissues and we hypothesize that PAI-1 expression is probably increased in patients' palmar tissue as well. Furthermore, its role may include inhibition of metalloproteinases (MMP). The MMP expression is indeed usually low in homeostasis (Clark et al., 2008) but high during extracellular matrix deposition. For example, it was recently found that MMP-14 silencing inhibits activation of proMMP-2 in fibroblasts isolated from affected DD tissues and that silencing of MMP-14 and MMP-2 genes may inhibit cell-mediated contractions (Wilkinson et al., 2012). Similarly, we observed elevated levels of TIMP-1 in TGF-β1 treated ND cells as well, which probably play a role in the inhibition of MMPs (Tuan et al., 2008). The balance between MMPs and TIMPs is critical for changes in the ECM and increased amounts of TIMPs have been previously observed in other fibrotic diseases (Chirco et al., 2006). For example, cords and nodules in DD have significantly increased levels of TIMP-1, TIMP-2, and MMP-2 gene expression of in comparison with control samples (Ulrich et al., 2009). Our results are consistent with these data as TGF-β1 increased the expression of TIMP-1 and MMP-2 both at the gene and protein levels which was visible as increased contractile force in the gel-contraction assay. This process was again reversed by inhibition of p38 phosphorylation.

In conclusion, our study provides additional evidence on the p38 MAPK signaling events in primary cells derived from DD patients' palmar fascia. In particular, TGF-β1-induced p38 phosphorylation in ND cells grown from macroscopically unaffected palmar fascia adjacent to the disease tissue of DD patients occurs with the activation of cytokine genes *CCL-11* and *IL-6* and secretion of extracellular matrix regulatory proteins PAI-1 and TIMP-1. Dual targeting of inflammation and p38 MAPK signaling in DD should be considered as a novel strategy for advancing the management of DD patients and prevention of disease recurrence.

## Ethical approval

All procedures performed in studies involving human participants were in accordance with ethical standards of the institutional and/or national research committee and with the 1964 Helsinki declaration and its later amendments or comparable ethical standards.

### Conflict of interest statement

The authors declare that the research was conducted in the absence of any commercial or financial relationships that could be construed as a potential conflict of interest.
