# Supplementary material for: Inflammatory Gene Expression Upon TGF-β1-Induced p38 Activation in Primary Dupuytren's Disease Fibroblasts
Source: Front Mol Biosci. 2015 Dec 8;2:68. doi: 10.3389/fmolb.2015.00068 (PMC4672058; doi:10.3389/fmolb.2015.00068)
Supplement: Supplementary file 1 [file DataSheet1.DOCX]

**Supplementary material: Genotyping results for IL-6 gene**

Maro Bujak^1^, Ivana Ratkaj^2^, Elitza Markova-Car^2^, Davor Jurišić^3^, Anita Horvatić^1^, Srđan Vučinić^1^, Jonatan Lerga^4,5^, Mirela Baus-Lončar^1^, Krešimir Pavelić^2^, Sandra Kraljević Pavelić ^2*^

**^1^ Ruđer Bošković Institute, Bijenička c. 54, 10000 Zagreb, Croatia**

**^2^ University of Rijeka, Department of Biotechnology, Radmile Matejčić 2, 51000 Rijeka, Croatia**

**^3^ University Hospital Centre Rijeka, Clinic for Surgery, Department for Plastic and Reconstructive Surgery, Krešimirova 42a, 51000 Rijeka, Croatia**

**^4^ University of Rijeka, Faculty of Engineering, Vukovarska 58, 51000 Rijeka, Croatia**

**^5^  Centre for advanced computing and modelling, University of Rijeka, Radmile Matejčić 2, 51000 Rijeka, Croatia**

**Material and methods**

**Genotyping for *IL-6* rs1800795 (-174 G/C), rs1800796 (-572 G/C) and rs1800797 (-597 A/G) polymorphisms**

Genomic DNA was extracted from peripheral blood collected from 44 patients and 46 control samples by QIAamp DNA Blood Mini Kit (Qiagen, Valencia, CA, USA), 200 µL of whole blood was used and DNA was eluted with 150 µL of elution buffer and concentration was measured on UV spectrophotometer. Genotyping for *IL-6* (-174 G/C), *IL-6* (-572 G/C) polymorphisms was performed on a real-time PCR with specific Dual-Labeled Fluorogenic Probes for allelic discrimination, created by Real-time PCR Assay Design Service (SIGMA-ALDRICH, St. Louis, Missouri, USA). All primers and probes were designed by Sigma Custom Oligonucleotide Synthesis and Custom Dual-Labeled Probe Synthesis services and purchased from Sigma (SIGMA-ALDRICH, St. Louis, Missouri, USA). Each oligonucleotide probe was labelled at the 5′ end with a different fluorescent reporter dye (FAM or Texas Red) to differentiate the amplification of each allele, and with a quencher dye (Black Hole Quencher 1 or Black Hole Quencher 2 respectively) at the 3′ end. All primers and probes used in the experiment are listed in Table 1.

The PCR cycling conditions were 95°C for 5 min, followed by 45 cycles of denaturation at 95°C for 15 s and annealing/extension at 58°C for 60 s. All DNA samples were run in triplicate. Obtained data were analyzed using the allelic discrimination analysis module available in the software of the iCycler iQ instrument (Bio-Rad, Foster-City, CA, USA). The normalized reporter signal was plotted onto X and Y axes, i.e., Texas Red and FAM reporter dyes, respectively. Analysis of *IL-6* (-597 G/A) polymorphism was performed with TaqMan Pre-designed SNP Genotyping Assay on a Applied Biosystems 7300 real-time PCR according to manufactures recommendation. The PCR cycling conditions were 95°C for 10 min, followed by 40 cycles of denaturation at 95°C for 15 s and annealing/extension at 60°C for 1 minute. Collected results were analyses with allelic discrimination analysis software available on Applied Biosystems 7300 PCR instrument. The frequency of the genotypes and alleles was evaluated using a *X^2^* test. The association was expressed as OR with 95% CI. The value of *p* less than 0.05 was considered statistically signiﬁcant.

**Table 1.** Primers and probes used for allelic discrimination analysis.

| Polymorphism | Primers and Probes |
| --- | --- |
| *IL-6* (-597) | rs 1800797 (Applied Biosystems, Foster City, CA, USA) |
| *IL-6* (-174) | Sense: 5-CTCAATGACGACCTAA GC-3  Antisense : 5-AATCTTTGTTGGAGGGTG-3  Probe 1 : 5-(6FAM)-TGTCTTGC C ATGCTAAAGGAC-(BHQ1)-3  Probe 2 : 5-(TxRd)-TGTCTTGC G ATGCTAAAGGAC-(BHQ2)-3 |
| *IL-6* (-572) | Sense: 5-CACGAAATTTGAGGATGG-3  Antisense: 5-GGATTATGAAGAAGGTAATACTA-3  Probe 1 : 5-(6FAM)-ACAACAGCC C CTCACAGG-(BHQ1)-3  Probe 2 : 5-(TxRd)-ACAACAGCC G CTCACAGG-(BHQ2)-3 |

**Results**

Analysis of gene expression in ND cells treated with TGF-β1 pointed to prominent up-regulation of *IL-6* as reported previously by Varjee et al. (Verjee et al., 2013). We therefore analysed well-defined polymorphisms in the promoter of *IL-6* gene -174 G/C, -597 G/A, -572 G/C which have been shown to affect both the transcription and secretion of *IL-6* in various diseases in a cohort of DD patients as a candidate for DD susceptibility (de-Madaria et al., 2008; Gordon et al., 2008). Allele and genotype frequencies of three *IL-6* polymorphisms were investigated in DD patients and in disease-free, control subjects (Table 2). All genotype frequencies analyzed were in Hardy–Weinberg equilibrium. The results showed no statistically significant (p>0.05) differences in the frequencies of *IL-6* polymorphisms genotypes and alleles between analysed groups. However, under the dominant model carriage of the A allele (GA/AA) significantly predisposes to DD in case of the -597 G/A polymorphism (P = 0.04, odds ratio [OR] 2.44, 95% confidence interval [95% CI] 1.01-5.9) (Table 2), suggesting that *IL-6* gene polymorphisms rs1800797 was associated with susceptibility to DD in the studied population.

**Linkage disequilibrium and haplotype analysis**

Further analysis of three polymorphisms in the *IL-6* gene cluster was done by Haploview. The LD (linkage disequilibrium) of SNPs and probable haplotypes with their frequencies (Table 3) have been calculated. Strong linkage disequilibrium was observed between *IL-6*, -597 G/A (rs1800797) and *IL-6*, -174 G/C (rs1800795). Haplotype analysis for relationship with affection status using Haploview found a significant association of AGG haplotype with DD (Table 3).

**Table 2.** Genotype and allele frequencies of three *IL-6* polymorphisms investigated in a cohort of DD patients in comparison with disease-free, control group (rs1800597, rs1800795, rs1800796).

| ***IL-6*** | **Patients n (%)** | **Controls n (%)** |
| --- | --- | --- |
| **rs1800797**  **(-597 G/A)** | GG = 12 (27)  GA = 23 (52)  AA = 9 (21) | GG = 22 (48)  GA = 14 (30)  AA = 10 (22) |
|  | ***Χ*^2^ = 5.141; *p* = 0.0765** | |
|  | G = 47 (53)  A = 41 (47) | G = 58 (63)  A = 34 (37) |
|  | ***Χ*^2^ = 1.718; *p* = 0.19; OR = 1.48, 95%CI = 0.82-2.69** | |
| Dominant model | GG= 12 (27)  GA/AA =32 (73) | GG= 22 (48)  GA/AA =24 (52) |
|  | **X^2^= 4.04; *p* = 0.04; OR = 2.44, 95%CI = 1.01-5.9** | |
| **rs1800795**  **(-174 G/C)** | GG = 12 (27)  GC = 23 (52)  CC = 9 (21) | GG = 21 (45)  GC = 15 (33)  CC = 10 (22) |
|  | ***Χ*^2^ = 4.149; *p* = 0.125** | |
|  | G = 47 (53)  C = 41 (47) | G = 57 (62)  C = 35 (38) |
|  | ***Χ*^2^ = 1.347; *p* = 0.245; OR = 1.42, 95%CI = 0.78-2.57** | |
| **rs180796**  **(-572 G/C)** | GG = 38 (86)  GC = 6 (14)  CC = 0 (0) | GG = 37 (80)  GC = 9 (20)  CC = 0 (0) |
|  | ***Χ*^2^ = 0.569; *p* = 0.450** | |
|  | G = 82 (93)  C = 6 (7) | G = 83 (90)  C = 9 (10) |
|  | ***Χ*^2^ = 0.5174; *p* = 0.47; OD = 0.67, 95%CI = 0.22-1.98** | |

**Table 3.** Probable frequencies of the common haplotypes in the IL-6 gene cluster in DD patients and screening disease-free, control cohorts.

| **Haplotype** | **Frequency** | **χ2** | **Case/Control ratio** | ***p* value** |
| --- | --- | --- | --- | --- |
| GGG | 0.454 | 2.515 | 0.394, 0.512 | 0.1127 |
| AGC | 0.343 | 0.99 | 0.379, 0.309 | 0.3197 |
| GGC | 0.064 | 1.043 | 0.084, 0.046 | 0.307 |
| GCG | 0.070 | 0.498 | 0.056, 0.083 | 0.4802 |
| AGG | 0.044 | 4.06 | 0.075, 0.014 | **0.0439^*^** |
| ACG | 0.015 | 0.506 | 0.009, 0.022 | 0.4769 |

**References**

**L. S. Verjee, J. S. N. Verhoekx, J. K. K. Chan, T. Krausgruber, V. Nicolaidou, D. Izadi et al. Unraveling the signaling pathways promoting fibrosis in Dupuytren's disease reveals TNF as a therapeutic target. P Natl Acad Sci USA2013. p. E928-E37.**

**de-Madaria, E., Martinez, J., Sempere, L., Lozano, B., Sanchez-Paya, J., Uceda, F., et al. (2008). Cytokine genotypes in acute pancreatitis: Association with etiology, severity,and cytokine levels in blood. *Pancreas.* 37, 295–301.**

**Gordon, A., Kiss-Toth, E., Stockley, I., Eastell, R., Wilkinson, J.M. (2008). Polymorphisms in the interleukin-1 receptor antagonist and interleukin-6 genes affect risk of osteolysis in patients with total hip arthroplasty. *Arthritis Rheum.* 58****, 3157–65.**
